# Lipoprotein(a) plasma levels are not associated with survival after acute coronary syndromes: An observational cohort study

**DOI:** 10.1371/journal.pone.0227054

**Published:** 2020-01-09

**Authors:** Christian Roth, Konstantin A. Krychtiuk, Clemens Gangl, Lore Schrutka, Klaus Distelmaier, Johann Wojta, Christian Hengstenberg, Rudolf Berger, Walter S. Speidl

**Affiliations:** 1 Division of Cardiology, Department of Internal Medicine II, Medical University of Vienna, Vienna, Austria; 2 Ludwig Boltzmann Cluster for Cardiovascular Research, Vienna, Austria; 3 Department of Internal Medicine I, Cardiology and Nephrology, Hospital of St. John of God, Eisenstadt, Austria; Erasmus Medical Center, NETHERLANDS

## Abstract

**Background:**

Lipoprotein(a) [Lp(a)] is associated with coronary artery disease in population studies, however studies on its predictive value in patients with cardiovascular disease, in particular after acute coronary syndromes (ACS), are conflicting. The aim of this study was to investigate whether Lp(a) is associated with survival after ACS.

**Methods and results:**

We analyzed Lp(a) measurement in 1,245 patients who underwent coronary angiography for ACS. The median follow-up for cardiovascular and all-cause mortality was 5.0 (IQR 3.2–8.0) years. 655 (52.6%) presented with ST-segment elevation myocardial infarction (STEMI), 424 (34.1%) with Non-ST-segment elevation myocardial infarction (NSTEMI) and 166 (13.3%) underwent coronary angiography for unstable angina. Cardiovascular mortality was 9.1% and all-cause mortality was 15.7%. Patients were stratified into four groups to their Lp(a) levels. (≤15mg/dL, >15-30mg/dL, >30-60mg/dL, and >60mg/dL). Multivessel disease was significantly more common in patients with Lp(a)>60mg/dL (p<0.05). Increased levels of Lp(a) were not associated with cardiovascular mortality (HR compared with Lp(a) ≤15mg/dL were 1.2, 1.2, and 1.0, respectively; p = 0.69) and not with all-cause mortality (HR compared with Lp(a) ≤15mg/dL were 1.2, 1.2, and 1.2, respectively; p = 0.46).

**Conclusions:**

Lp(a) levels at time of ACS were neither associated with cardiovascular nor with all-cause mortality. Although Lp(a) has been shown to be associated with incidence of coronary artery disease, this study does not support any role of Lp(a) as a risk factor for mortality after ACS. This should be taken into account for development of outcome studies for agents targeting Lp(a) plasma levels.

## Introduction

Cardiovascular disease is the leading cause of death in industrial countries, with coronary heart disease (CHD) being the most common reason of death [1]. In recent years, epidemiologic and mendelian randomization studies have established Lipoprotein(a) [Lp(a)] as an independent risk factor for atherosclerosis and cardiovascular events in general population studies [2]. Lp(a) is a lipoprotein that contains similar to low density lipoprotein (LDL) apolipoprotein B and in addition apolipoprotein(a) which is attached by a disulfide bridge [3]. Increased Lp(a) plasma concentrations have been associated with increased risk of CHD, interacting with, but independently of other risk factors [4–6]. Recently, a large meta-analysis including 18 general population studies showed a risk for incident CHD for people with elevated Lp(a) levels [7]. However, studies on the role of Lp(a) in patients with established CHD, in particular in patients with acute coronary syndromes (ACS) are conflicting [8]. An analysis of the Ludwigshafen Risk and Cardiovascular Health (LURIC) study that enrolled patients without ACS who underwent coronary angiography (prevalence of CHD at baseline 78%), was not able to establish an association between Lp(a) levels and mortality [9]. Recently, a nested case-cohort analysis of the dal-Outcomes trial that investigated the effect of dalcetrapib in patients after ACS, found no association of elevated Lp(a) with adverse outcome [10]. Similarly, an analysis of the Pravastastin or Atorvastatin Evaluation and Infection Therapy (PROVE-IT) trial that included patients stabilized after a recent ACS, was not able to establish an association between Lp(a) plasma levels and the occurrence of major cardiovascular event [11]. In contrast, a recent meta-analysis of statin outcome trials showed that baseline and on-statin Lp(a) levels were independent, linear related with cardiovascular outcome [12]. Beside primary prevention studies, this analysis included data from Long-Term Intervention with Pravastatin in Ischaemic Disease (LIPID), Myocardial Ischemia Reduction with Acute Cholesterol Lowering (MIRACL) and Scandinavian Simvastatin Survival Study (4S) that all randomized patients with previous myocardial infarction or unstable angina in a stable phase of the disease. Trial-specific hazard ratios showed a positive, albeit weak association between Lp(a) and outcome also in these secondary prevention studies, respectively. However, it has to be mentioned that patients in these studies were included between 1990 and 1999, with low rates of acute revascularization procedures and without contemporary secondary prevention. Recently, a retrospective analysis of the Further Cardiovascular Outcomes Research with PCSK9 Inhibition in Subjects with Elevated Risk (FOURIER) Trial showed that in patients with cardiovascular disease (81.1% history of myocardial infarction, blood sampling median 3.3 years after the event) Lp(a) levels in the highest quartile are associated with major cardiovascular events but not with increased cardiovascular mortality.[13, 14]

So far it has been challenging to modify Lp(a) levels by lifestyle or medical interventions. Although proprotein convertase subtilisin/kexin type 9 (PCSK9) inhibitors can lower Lp(a) by 20% to 30% in the total cohort, these effects are lower in patients with substantially elevated levels [15, 16]. Lipoprotein apheresis can be used to lower Lp(a) significantly, however this approach is costly, time and resource consuming. Using a novel approach employing antisense oligonucleotide inhibitors of apolipoprotein(a) a new treatment that could lower Lp(a) by 90% is on the horizon [17]. In this context, it is of utmost interest whether Lp(a) is a strong risk factor for patients with established CHD, in particular in secondary prevention patients after ACS.

Therefore, the aim of this study was to investigate whether Lp(a) is associated with survival in patients after ACS.

## Methods

### Study population

This single-center observational cohort study selected patients from the coronary catheter laboratory database of the Medical University of Vienna (CCLD-MUW) between the years 2004 to 2012 who underwent coronary angiography for ACS. Experienced interventionists approved all coronary angiograms according to contemporary guidelines. The study is in line with the Declaration of Helsinki and was approved by the ethics committee of the Medical University of Vienna.

### Data collection

The CCLD-MUW is a comprehensive database, which had been established in co-operation with the informatics department of the MUW gathering all patients from the catheter laboratory database of the MUW. Study data includes baseline characteristics, co-morbidities, angiographic characteristics and blood results.

The survival status including date and mode of death of all patients in the database was prospectively retrieved from the Austrian death registry database (Statistik Austria) at the due day (December 31^st^, 2014).

### Measurement of Lp(a)

Lp(a) was measured in the clinical routine by an isoform-insensitive immunonephelometric assay (Roche Diagnostics, Germany; intra-assay coefficient of variation ranging from 0.9 to 6.2% and inter-assay coefficient of variation ranging from 5.1 10 10.9) [18].

### Statistical analysis

Sample size calculation revealed that in a cohort with a cardiovascular mortality rate of 9%, given a power of 0.8 and a significance level of 0.05, we would need 1240 patients to detect a difference of 25% in Lp(a) levels between patients with or without cardiovascular death. Categorical variables are summarized as counts or percentages and are compared by the χ2 or by Fisher’s exact test as appropriate. Continuous variables are expressed as median (interquartile range). Unpaired variables were compared by Mann-Whitney test for two samples and by Kruskal-Wallis test for multiple samples. Paired variables were compared by Wilcoxon rank-sum test. Cox proportional hazard regression analysis was performed to assess the effect of Lp(a) on survival. Hazard ratios (HR) per standard deviation (SD) were calculated after log-transformation. In addition, patients were stratified according their Lp(a) levels in four groups (≤15mg/dL, >15-30mg/dL, >30-60mg/dL, and >60mg/dL). The cut-off of >60mg/dL was chosen as this is the cut-off for Lp(a)-apheresis according German guidelines [19]. Kaplan–Meier analysis (log-rank test) was applied to verify the time-dependent discriminative power of Lp(a) categories. Two-sided p-values of <0.05 indicated statistical significance. SPSS 22.0 (IBM Corporation, Armonk, NY, USA) was used for all analyses.

## Results

We analyzed Lp(a) measurement in 1,245 patients who underwent coronary angiography for ACS, with a median follow-up time of 3.2 (IQR 5.0–8.1) years. Baseline characteristics are given in Table 1. The median age of patients was 60.3 (IQR 51.0–70.2) years and 939 (75.4%) were male. 655 (52.6%) had a STEMI, 424 (34.1%) were admitted for NSTEMI and 166 (13.3%) underwent coronary angiography for unstable angina. Patients were stratified into four groups according to their Lp(a) levels (≤15mg/dL, >15-30mg/dL, >30-60mg/dL, and >60mg/dL).

**Table 1:** Baseline characteristics.

	Lp(a) ≤15mg/dL n = 195	Lp(a) 15-30mg/dL n = 362	Lp(a) >30-60mg/dL n = 319	Lp(a) >60mg/dL n = 369	p-value
**Lp(a)** (mg/dL), median (IQR**)**	11 (11–13)	21 (17–25)	39 (33–49)	99 (78–134)	
**Age** (years), median (IQR)	63 (52–71)	61 (51–70)	60 (51–71)	59 (50–69)	0.446
**Male gender**, n (%)	153 (79)	279 (77)	236 (74)	271 (73)	0.450
**Hypertension**, n (%)	160 (82)	265 (73)	224 (70)	266 (72)	0.024
**Diabetes Mellitus**, n (%)	46 (24)	77 (21)	74 (23)	74 (20)	0.693
**Current smoker**, n (%)	81 (42)	179 (49)	143 (45)	160 (43)	0.239
**Multivessel disease**, n (%)	113 (57)	193 (53)	170 (53)	229 (62)	0.018
**Statin treatment**, n (%)	177 (91)	330 (91)	287 (90)	341 (92)	0.725
**Family history of CHD**, n (%)	53 (27)	98 (27)	77 (24)	108 (29)	0.514
**BMI** (kg/m^2^), median (IQR)	28 (25–31)	27 (25–31)	27 (25–30)	27 (24–31)	0.530
**HbA1c** (%), median (IQR)	5.9 (5.5–6.3)	5.8 (5.5–6.3)	5.8 (5.5–6.4)	5.8 (5.5–6.2)	0.662
**Creatinine** (mg/dL), median (IQR)	1.00 (0.89–1.13)	1.03 (0.89–1.16)	1.01 (0.88–1.21)	1.04 (0.91–1.21)	0.708
**Triglycerides** (mg/dL), median (IQR)	155 (104–227)	137 (93–195)	136 (187–191)	129 (92–201)	0.021
**Total cholesterol** (mg/dL), median (IQR)	193 (163–227)	202 (172–233)	202 (168–235)	210 (181–247)	<0.001
**HDL** (mg/dL), median (IQR)	43 (37–50)	42 (36–50)	43 (35–51)	44 (37–51)	0.310
**LDL** (mg/dL), median (IQR)	115 (88–146)	130 (99–158)	128 (93–155)	136 (107–164)	<0.001
**CRP** (mg/dL), median (IQR)	0.46 (0.24–1.15)	0.52 (0.25–1.25)	0.54 (0.24–1.44)	0.58 (0.25–1.30)	0.656

Lp(a) Lipoprotein(a); BMI body mass index; CHD coronary artery disease; CRP C-reactive protein; HDL high density lipoprotein; LDL low density lipoprotein.

Patients with higher Lp(a) levels showed significant lower plasma levels of triglycerides and higher levels of total cholesterol and LDL cholesterol (Table 1). Hypertension was less prevalent in patients with high levels of Lp(a) and patients with Lp(a)>60mg/dL had significantly more often multivessel disease (62.1% vs 55.5%; p<0.05). Median follow-up for cardiovascular and all-cause mortality was 5.0 (IQR 3.2–8.0) years. Cardiovascular mortality was 9.1% and all-cause mortality was 15.7%.

**Fig 1:**
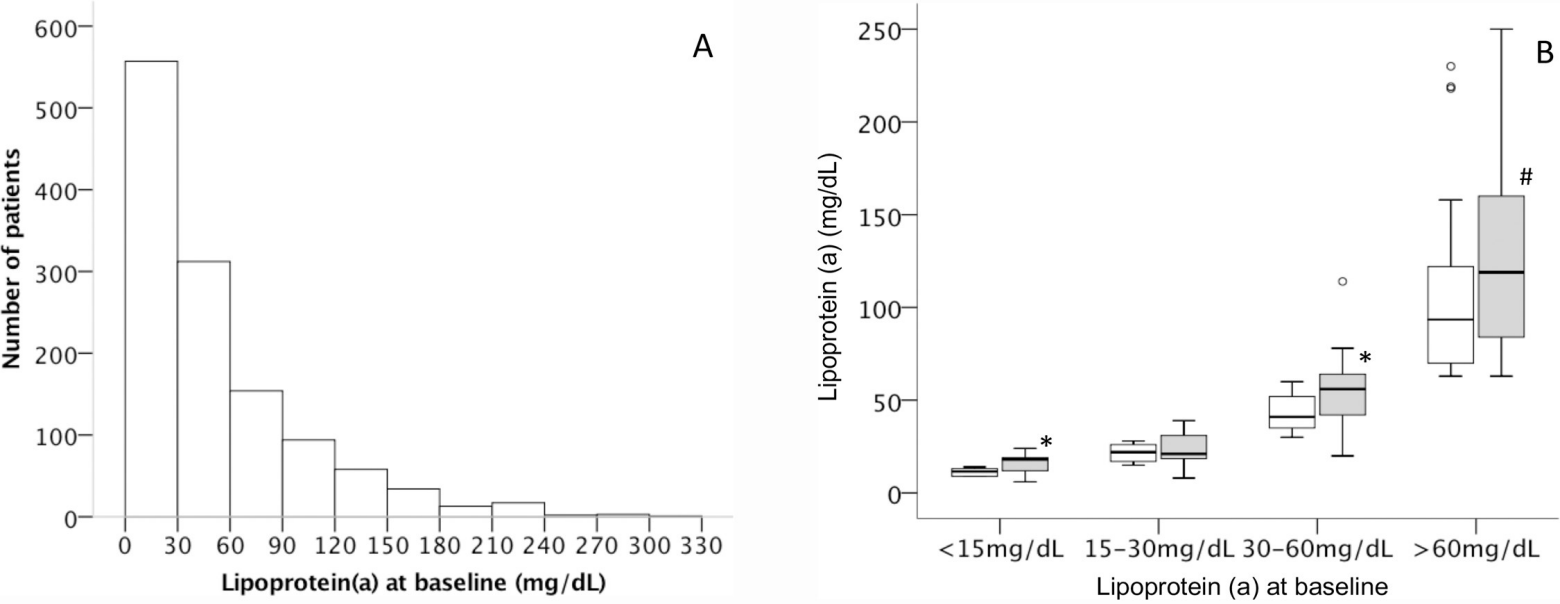
Lipoprotein (a) plasma levels at baseline and change of lipoprotein (a) plasma levels over time between acute coronary syndrome and one to three months follow-up. Plasma levels of lipoprotein(a) were measured at time of acute coronary syndrome (A). In a subgroup of 80 patients (B) lipoprotein (a) was measured at baseline (white boxes) and after one to three months of follow-up (grey boxes). Patients were stratified in four groups according baseline Lp(a) levels: ≤15mg/dL, >15-30mg/dL, >30-60mg/dL, and >60mg/dL; * p<0.05, # p<0.001 baseline vs. follow-up.

In a subgroup of 80 patients Lp(a) was measured twice, at baseline and after one to three months. Median Lp(a) slightly increased overtime from 39 (IQR 20–70) mg/dL to 43 (20–86) mg/dL (p<0.001). This increase was present in patients with low Lp(a)-values of ≤15mg/dL and in patients with higher Lp(a)-values of >30mg/dL, but not in patients with values of >15-30mg/dL (Fig 1). However, plasma levels of Lp(a) at baseline and after one to three months showed a very high correlation (R = 0.94; p<0.001).

**Fig 2:**
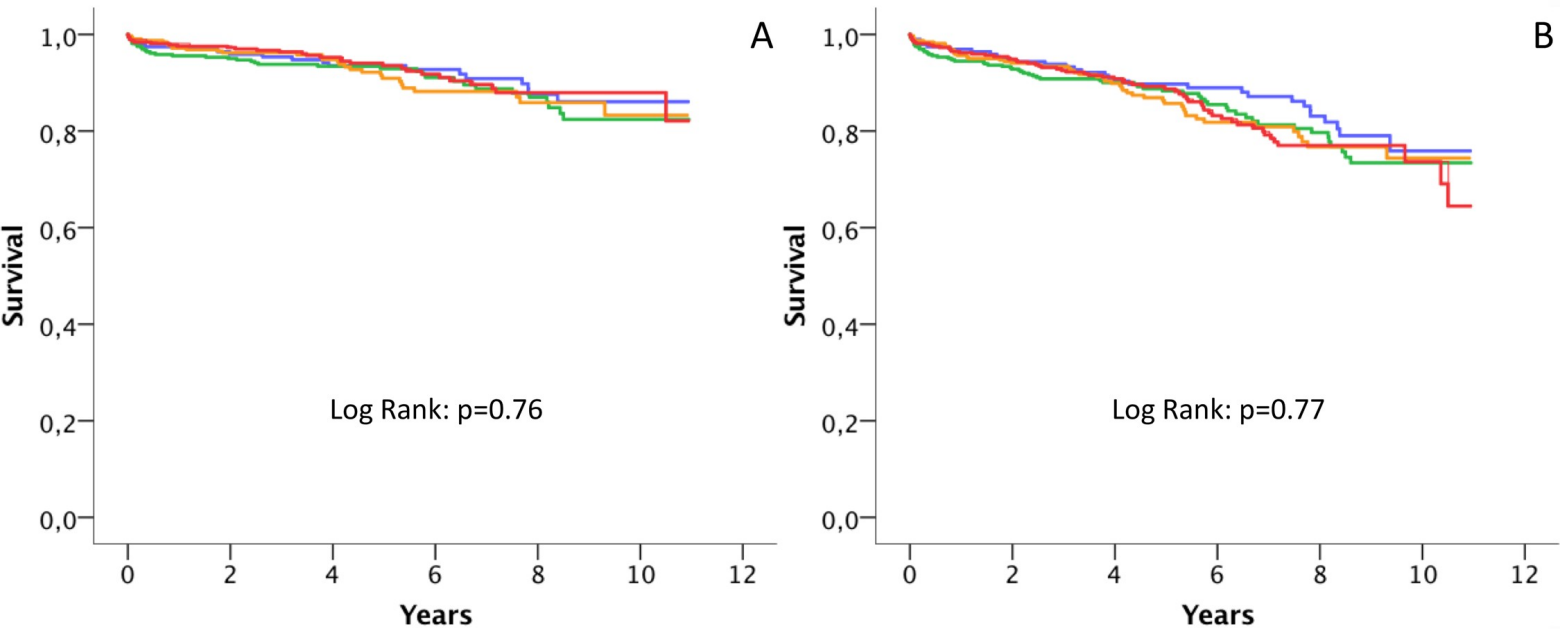
**Kaplan-Meier survival curves according categories of lipoprotein (a).** Patients were stratified in four groups according Lp(a) levels: ≤15mg/dL (blue), >15-30mg/dL (green), >30-60mg/dL (orange), and >60mg/dL (red). Kaplan-Meier survival curves for cardiovascular mortality (A) and all-cause mortality (B).

Median Lp(a) levels were not different in patients with (31 IQR 17–60 mg/dL) or without cardiovascular death (33 IQR 18–73 mg/dL; p = 0.29) or in patients with (32 IQR 17–76 mg/dL) or without death of any cause (32 IQR 18–17 mg/dL; p = 0.70), respectively. Log-transformed Lp(a) levels were not associated with cardiovascular mortality (HR per SD 0.96, 95% CI 0.80–1.15; p = 0.63) or all-cause mortality (HR per SD 1.02, 0.89–1.17; p = 0.76). In addition, Lp(a) groups (≤15mg/dL, >15-30mg/dL, >30-60mg/dL, and >60mg/dL) were not associated with cardiovascular mortality (Fig 2A) or all-cause mortality (Fig 2B). The HR for cardiovascular mortality compared with the group of patients with Lp(a) ≤15mg/dL were 1.2 (95% CI 0.7–2.1), 1.2 (95% CI 0.6–2.1), and 1.0 (95% CI 0.5–1.7) for patients with Lp(a) plasma levels of >15-30mg/dL, >30-60mg/dL, and >60mg/dL, respectively (p = 0.69). The HR for all-cause mortality compared with the group of patients with Lp(a) ≤15mg/dL were 1.2 (95% CI 0.8–1.9), 1.2 (95% CI 0.8–2.0), and 1.2 (95% CI 0.8–1.9) for patients with Lp(a) plasma levels of >15-30mg/dL, >30-60mg/dL, and >60mg/dL, respectively (p = 0.46). Multivariate analysis that included age, sex, diabetes, hypertension, BMI, LDL-cholesterol, triglycerides, presence of multivessel disease and type of event (STEMI, NSTEMI or unstable angina) showed similar results (Table 2).

**Table 2:** Multivariate analyses of association between lipoprotein (a) and cardiovascular or total mortality after acute coronary syndromes.

Lp(a) levels	Hazard ratio	95% CI	p-value
**Cardiovascular mortality**			
Unadjusted			
≤15mg/dL	1.0		
>15-30mg/dL	1.2	0.7–2.1	0.50
>30-60mg/dL	1.2	0.6–2.1	0.82
>60mg/dL	1.0	0.5–1.7	0.67
**Adjusted for age, sex, diabetes, hypertension, BMI, LDL-cholesterol, triglycerides, presence of multivessel disease and type of event (STEMI, NSTEMI or unstable angina)**			
≤15mg/dL	1.0		
>15-30mg/dL	1.1	0.6–2.1	0.75
>30-60mg/dL	1.0	0.5–2.0	0.98
>60mg/dL	1.1	0.6–2.0	0.84
**Total mortality**			
Unadjusted			
≤15mg/dL	1.0		
>15-30mg/dL	1.2	0.8–1.9	0.34
>30-60mg/dL	1.2	0.8–2.0	0.35
>60mg/dL	1.2	0.8–1.9	0.36
**Adjusted for age, sex, diabetes, hypertension, BMI, LDL-cholesterol, triglycerides, presence of multivessel disease and type of event (STEMI, NSTEMI or unstable angina)**			
≤15mg/dL	1.0		
>15-30mg/dL	1.1	0.7–1.8	0.64
>30-60mg/dL	1.1	0.7–1.8	0.75
>60mg/dL	1.2	0.7–1.9	0.55

BMI denotes body mass index

**Fig 3:**
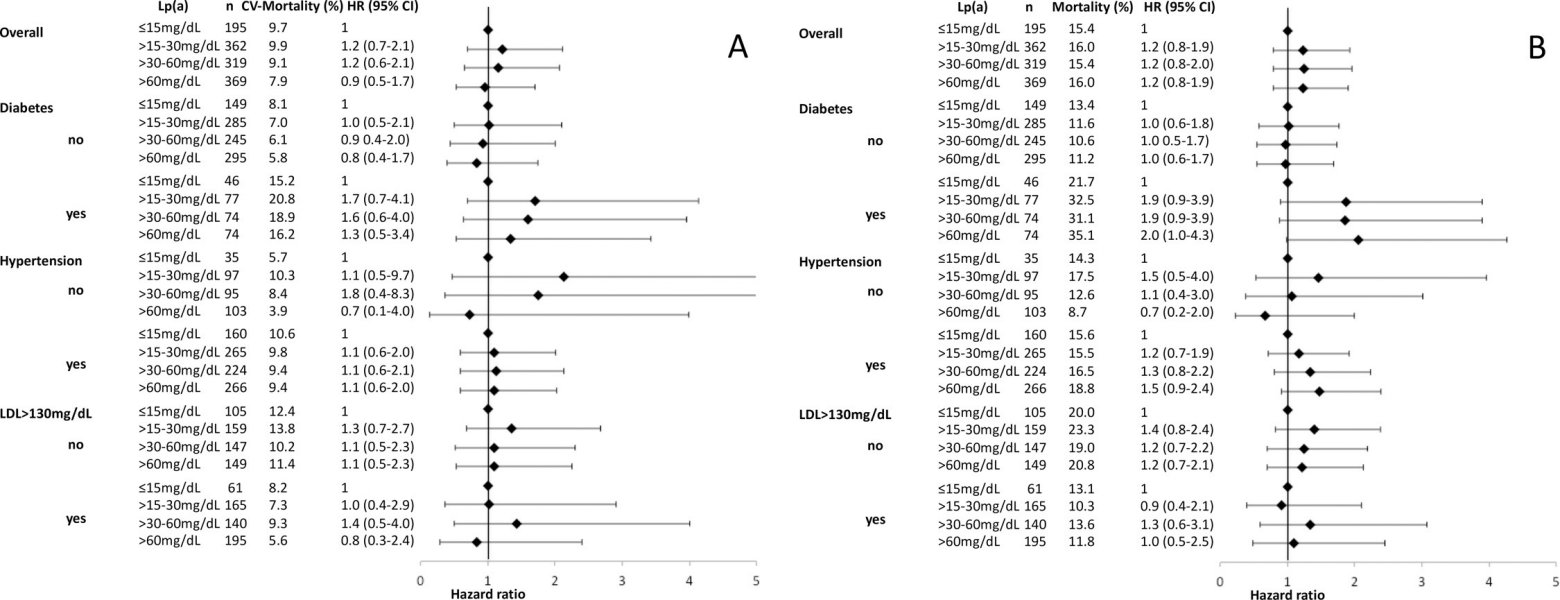
Forest plots. Hazard ratios for Lp(a) levels for cardiovascular mortality (A) and all-cause mortality (B) according the presence of diabetes, hypertension and LDL cholesterol below or above the median (130mg/dL).

When patients were stratified according to the presence of diabetes, hypertension or median level of LDL cholesterol (130mg/dL), results were comparable (Fig 3). The only subgroup with a borderline trend to increased risk was diabetic patients with Lp(a)>60mg/dL (HR for all-cause mortality compared to diabetic patients with Lp(a) ≤15mg/dL: 2.05, 95% CI 0.99–4.27; p = 0.054).

Patients with Lp(a) above the 90th percentile (>120mg/dL) did not have an increased risk for cardiovascular mortality (HR 1.14, 95% CI 0.63–2.08; p = 0.80) and all-cause mortality (HR 1.10, 95% CI 0.69–1.73; p = 0.71) as compared to patients with Lp(a) levels ≤120mg/dL.

In patients with ACS, early mortality may be mainly due to thrombotic events or due to heart failure, respectively. Therefore, we performed two landmark analyses for late mortality in patients that survived the first 30 days (n = 1228) or the first year (n = 1191), respectively. Log-transformed Lp(a) levels were not associated with cardiovascular mortality (HR per SD 0.97, 95% CI 0.80–1.18; p = 0.79) or all-cause mortality (HR per SD 1.03, 95% CI 0.89–1.19; p = 0.68) in patients that survived the first 30 days nor with cardiovascular mortality (HR per SD 1.01, 95% CI 0.81–1.26; p = 0.95) or all-cause mortality (HR per SD 1.05, 95% CI 0.89–1.23; p = 0.58) in patients that survived the first year after ACS.

## Discussion

In this study, including 1245 patients after ACS, plasma levels of Lp(a) were not associated with cardiovascular and all-cause mortality, respectively. This is in line with two recent studies that specifically included post-ACS patients from two randomized controlled trials. In the PROVE-IT trial Lp(a) was measured approximately one week [11] and in the dal-Outcomes trial Lp(a) was measured 4 to 12 weeks after ACS [10]. In contrast to the two previous studies that included patients from randomized controlled trials for lipid modifying agents, our study is, to our knowledge, the first analysis of an all-comers post-ACS registry that investigated the association between Lp(a) plasma levels and survival.

In our study Lp(a) was measured one to three days after the acute event. Therefore, acute phase reaction could have impacted the association between Lp(a) and outcome. To address this, we have analyzed a subgroup of 80 patients in which Lp(a) was measured twice, at baseline and after one to three months. Although Lp(a) increased minimally over-time, correlation between baseline and follow-up was high. Therefore, it is unlikely that the neutral results are caused by the time of Lp(a)-measurement. In this study, we have chosen a cut-off of 60 mg/dL for the highest risk group, as this is the value that is the indication for lipoprotein apheresis for elevated Lp(a) in patients with repeat CHD events despite optimal LDL-lowering therapy in Germany [19].

Interestingly, Lp(a) levels in this cohort are relatively high (median 32mg/dL) as compared to previously reported levels (median range 5-17mg/dL) [10]. However, this in line with a different Austrian cohort (Zitiat graz) [20]. In contrast to most previous studies, in this study Lp(a) was measured at time of blood sampling and not after storage of plasma samples. It has been shown that Lp(a) can decline by 7% within a storage time of 2 years [21], which could have been a confounder in previous studies with long-time storage of plasma samples before analysis. In addition, the long-time storage in previous studies could be one factor why median Lp(a) levels in this study are higher than previously reported levels [10].

Multivessel disease was more prevalent in patients with elevated levels of Lp(a). This is in line with results from the LURIC study [9] and supports mendelian randomization [22] and population based studies [7] that showed an increased prevalence and a more severe disease in subjects with elevated levels of Lp(a).

Lp(a) levels show a skewed distribution and very high levels of Lp(a) have been associated with an increased incidence of atherosclerosis [15]. Therefore, we analyzed whether very high plasma levels of Lp(a) > the 90^th^ percentile are associated with outcome. Interestingly, also patients with plasma levels of Lp(a) >120 mg/dL did not show an increased risk for cardiovascular or all-cause mortality. However, due to the smaller group of patients statistical power for this analysis is decreased.

When patients were stratified in subgroups according to the presence of diabetes, hypertension or median level of LDL cholesterol we also could not detect an association between Lp(a) and cardiovascular and all-cause mortality. Only in the subgroup of patients with diabetes, Lp(a) >60mg/dL showed a borderline association with all-cause mortality. However, this has to be taken with caution, as this is an exploratory retrospective subgroup-analysis and was not adjusted for multiple comparisons. In addition, these results are in contrast to a previous study that showed an association between Lp(a) in non-diabetic but not in diabetic patients [23].

One possible factor that might contribute to the neutral results of this study is the index event bias [24]. Patients with high Lp(a) levels show paradoxically lower levels of triglycerides and a lower incidence of hypertension. This could be due to the fact, that patients with elevated Lp(a) as a genetic risk factor develop CHD and an ACS despite a lower prevalence of other risk factors. This uneven distribution of risk factors could increase the risk of patients with low Lp(a) for further events [8]. However, multivariate adjustment for multiple risk factors did not change the association between plasma levels of Lp(a) and mortality.

### Limitations

Three major limitations of this study have to be taken into account. First, this is a single center register and the results have to be confirmed be other centers. However, our results are in line with other studies that included patients with established atherosclerosis as discussed above. In addition, our endpoints are cardiovascular and all-cause mortality and we do not have data on other cardiovascular endpoints like stroke, myocardial infarction or revascularization procedures. In addition, Lp(a) levels are well known to be determined by apo(a) size, where smaller sizes are associated with higher Lp(a) levels. In this study, Lp(a) levels were determined directly after blood sampling using a routine assay and blood samples of this cohort were not stored. Therefore, no data are available regarding apo(a) size which could have influenced our results.

## Conclusion

Lp(a) levels at time of ACS were not associated with cardiovascular or all-cause mortality. Although Lp(a) has been shown to be associated with incidence of coronary artery disease, this study does not support any role of Lp(a) as risk factor for mortality after ACS. This should be taken into account for the development of outcome studies for agents targeting Lp(a) plasma levels.

## Supporting information

S1 File (XLSX)
